# Diverse BCR usage and T cell activation induced by different COVID-19 sequential vaccinations

**DOI:** 10.1128/mbio.01429-24

**Published:** 2024-09-09

**Authors:** Junxiang Wang, Kaiyi Li, Yuan Wang, Zhengfang Lin, Weidong Li, Jinpeng Cao, Xinyue Mei, Rui Wei, Jinglu Yang, Xiaobing Zhai, Deyi Huang, Kaiwen Zhou, Xinyue Liang, Zhongfang Wang

**Affiliations:** 1 State Key Laboratory of Respiratory Disease and National Clinical Research Center for Respiratory Disease, Guangzhou Institute of Respiratory Health, the First Affiliated Hospital of Guangzhou Medical University, Guangzhou Medical University, Guangzhou, China; 2 Department of Clinical Laboratory, Dongguan Maternal and Child Health Care Hospital, Dongguan, China; 3 Guangzhou National Laboratory, Bioland, Guangzhou, Guangdong, China; 4 West China Hospital, West China Medical School, Sichuan University, Chengdu, Sichuan, China; 5 Shenzhen Hetao Institute, Guangzhou National Laboratory, Shenzhen, Guangdong, China; Monash University, Clayton, Victoria, Australia

**Keywords:** SARS-CoV-2, vaccine, BCR usage, T cells

## Abstract

Limited knowledge is available on the differences in severe acute respiratory syndrome coronavirus 2 (SARS-CoV-2) specific antibody breadth and T cell differentiation among different COVID-19 sequential vaccination strategies. In this study, we compared the immunogenicity of the third different dose of COVID-19 vaccines, such as mRNA (I-I-M), adenoviral vector (I-I-A), and recombinant protein (I-I-R) vaccines, in terms of the magnitude and breadth of antibody response and differentiation of SARS-CoV-2-specific T and B cells. These studies were performed in the same clinical trial, and the samples were assessed in the same laboratory. IGHV1-69, IGHV3-9, and IGHV4-34 were the dominant B cell receptor (BCR) usages of the I-I-M, I-I-A, and I-I-R groups, respectively; the RBD^+^ B cell activation capacities were comparable. Additionally, the I-I-R group was characterized by higher numbers of regulatory T cells, circulating T follicular helper cells (cTFH) – cTFH1 (CXRC3^+^CCR6^-^), cTFH1-17 (CXRC3^+^CCR6^+^), cTFH17 (CXRC3^-^CCR6^+^), and cTFH-CM (CD45RA^-^CCR7^+^), and lower SMNE^+^ T cell proliferative capacity than the other two groups, whereas I-I-A showed a higher proportion and number of virus-specific CD4^+^ T cells than I-I-R, as determined in ex vivo experiments. Our data confirmed different SARS-CoV-2-specific antibody profiles among the three different vaccination strategies and also provided insights regarding BCR usage and T/B cell activation and differentiation, which will guide a better selection of vaccination strategies in the future.

**IMPORTANCE:**

Using the same laboratory test to avoid unnecessary interference due to cohort ethnicity, and experimental and statistical errors, we have compared the T/B cell immune response in the same cohort sequential vaccinated by different types of COVID-19 vaccine. We found that different sequential vaccinations can induce different dominant BCR usage with no significant neutralizing titers and RBD^+^ B-cell phenotype. Recombinant protein vaccine can induce higher numbers of regulatory T cells, circulating TFH (CTFH)1, CTFH17, and CTFH-CM, and lower SMNE^+^ T-cell proliferative capacity than the other two groups, whereas I-I-A showed higher proportion and number of virus-specific CD4^+^ T cells than I-I-R. Overall, our study provides a deep insight about the source of differences in immune protection of different types of COVID-19 vaccines, which further improves our understanding of the mechanisms underlying the immune response to SARS-CoV-2.

## INTRODUCTION

Since the coronavirus disease 2019 (COVID-19) outbreak, severe acute respiratory syndrome coronavirus 2 (SARS-CoV-2) has caused a widespread pandemic, posing considerable challenges to global public health and safety (1, 2). Multiple variants of SARS-CoV-2 with higher transmission capacity and improved immune escape capacity have since emerged (3, 4). To effectively prevent SARS-CoV-2 infection, different types of SARS-CoV-2 vaccines have been successively developed worldwide using various technical strategies based on the characteristics of the virus, such as the inactivated vaccine from Kexing Biopharm in China (CoronaVac), the mRNA vaccine from Moderna and Pfizer (mRNA-1273 and BNT162b2, respectively), the adenoviral vector vaccine from AstraZeneca (ChAdOx1), and the recombinant protein vaccine from Zhifei Biological and Novavax (ZF2001 and NVX-CoV2373) (5–9). Although the protective efficacy of the COVID-19 vaccines varies, they effectively protected against disease severity and fatality. Many studies have already reported the immune potency of different types of vaccines produced via varying technical routes in different cohorts worldwide (10). However, few studies have compared the same cohort and tested the immune response using the same laboratory test to avoid unnecessary interference, such as race errors in cohorts, errors in experimental manipulation, and statistical errors in the experimental data (11–13).

Different types of vaccines, such as mRNA, adenoviral vectors, and recombinant vaccines, can initiate different patterns of antigen presentation. mRNA vaccines are synthesized *via in vitro* transcription, which directs protein translation in the cytoplasm to produce viral antigen(s) *in vivo*. In adenoviral vector vaccines, gene(s) encoding pathogen antigens(s) are cloned into non-replicating or replicating vectors, and the antigen(s) are produced by the transduced host cells after immunization. Recombinant protein vaccines comprise key viral proteins or peptides that can be manufactured *in vitro* using bacterial, yeast, insect, or mammalian cells (14). However, some characteristics of different types of COVID-19 vaccines should be noted; for example, antigen processing of the mRNA vaccine is similar to the processing of the spike (S) protein in the virus infection cycle; the spike protein displayed in adenoviral vector vaccines can possibly be influenced by the vector proteins; and the recombinant protein vaccine is dependent on their designed sequence and structure. Our previous data showed that the third dose of heterogenous booster (mRNA, adenoviral vector, and recombinant protein) based on two inactivated vaccines enhanced not only the level of the Nabs in magnitude and breadth, but also the T cell response (15, 16). However, the underlying mechanism is unknown. In this study, we aimed to study the relevant immune mechanisms.

Here, we recruited a cohort receiving intramuscularly administered mRNA, adenoviral vector, and recombinant COVID-19 vaccines developed by Walvax Biotechnology Co., Ltd after two doses of inactivated vaccines (17) to compare the immune response, including factors, such as the magnitude and differentiation of T- and B-cell responses, at the same time points following the third dose of vaccination in the same laboratory. Our observations aim to identify a better vaccination strategy, provide a reference for the formulation of new epidemic prevention strategies, and contribute in-depth information on the SARS-CoV-2 immune response.

## RESULTS

### Titers of anti-S protein IgG antibody and neutralizing antibody against WT in the I-I-M group were higher than those in the I-I-A group

**Fig 1:**
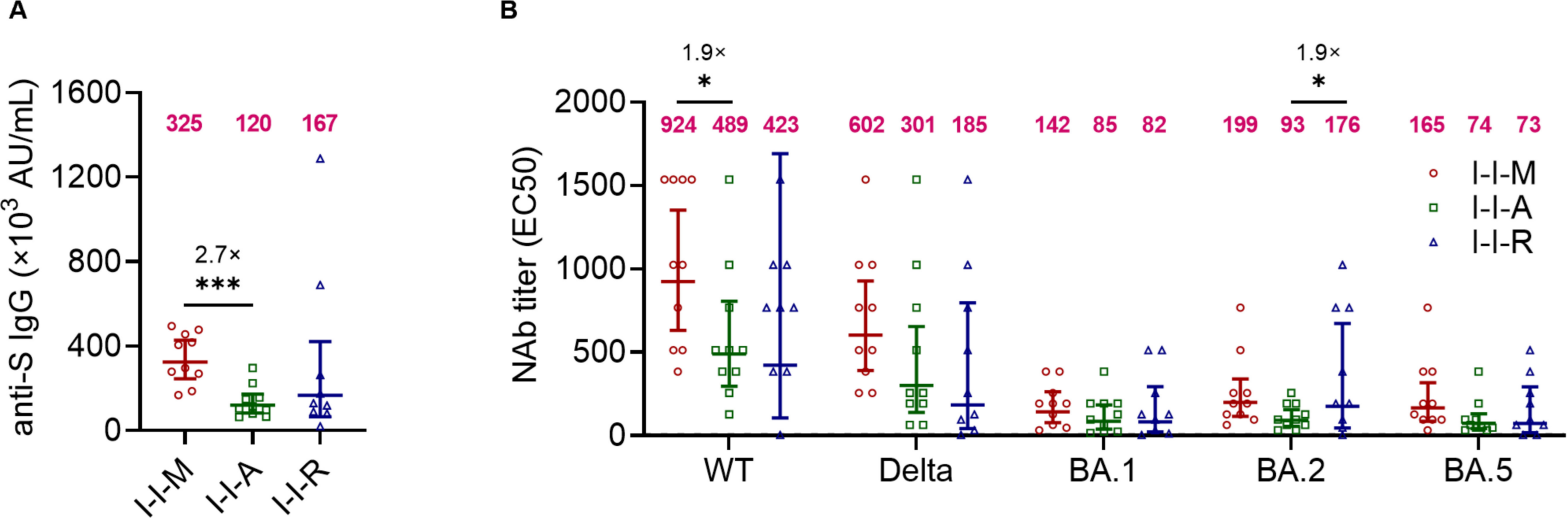
Comparison of anti-S IgG titers and Nab titers against WT, Delta, BA.1, BA.2, and BA.5 among I-I-M, I-I-A, and I-I-R among the I-I-M, I-I-A, and I-I-R groups. (A) The antibody titer of anti-S IgG among I-I-M, I-I-A, and I-I-R. (B) Comparison of Nab titers against WT, Delta, BA.1, BA.2, and BA.5 among I-I-M, I-I-A, and I-I-R. The numbers in magenta indicate the geometric mean titers (GMTs), and fold change in GMT for the virus compared with the strain for which the neutralizing titer is lower in A-B. Significance was measured using the Mann‒Whitney test. *P < 0.05, **P < 0.01, ***P < 0.001. I-I-M, people receiving one dose of mRNA vaccine 14 days after two doses of inactivated vaccine (*n* = 10); I-I-A, people receiving one dose of Adv vector vaccine 14 days after two doses of inactivated vaccine (*n* = 10); I-I-R, people receiving one dose of recombinant protein vaccine 14 days after two doses of inactivated vaccine (*n* = 9).

To investigate the differences among the three groups (I-I-M, I-I-A, and I-I-R) in inducing SARS-CoV-2-specific humoral immune responses, we first examined S-specific antibody titers 14 days after the third dose of vaccination. The titers of anti-S IgG antibodies against I-I-M, I-I-A, and I-I-R were 325,000, 120,000, and 167,000 AU/mL, respectively. I-I-M induced titers significantly higher than that of I-I-A (325,000 vs 120,000, 2.7-fold) (Fig. 1A). Next, we tested the neutralization of WT, Delta, BA.1, BA.2, and BA.5 variants in the three groups. I-I-M, I-I-A, and I-I-R induced Nab titers against WT, Delta, BA.1, BA.2, and BA.5 variants. Of these, the I-I-M-induced Nab titers of WT, Delta, BA.1, BA.2, and BA.5 were 924, 602, 142, 199, and 165, whereas those induced by I-I-A were 489, 301, 85, 93, and 74, and by I-I-R were 423, 185, 82, 176, and 73, respectively Fig. S1A; Tables S2 to S4). Further analysis showed that both I-I-M and I-I-A had significantly higher WT and Delta Nab titers than BA.1, BA.2, and BA.5 Nab titers. In contrast, only the WT Nab titer of I-I-R was significantly higher than the BA.1 and BA.5 Nab titers. Notably, despite the lack of any significant difference, the BA.2 Nab titers of I-I-M, I-I-A, and I-I-R were 1.4-fold, 1.1-fold, and 2.1-fold higher than those of BA.1, respectively (Fig. S1B through D). However, I-I-M showed a significantly higher titer of WT Nab than I-I-A (924 vs 489, 1.9-fold), and the BA.2 Nab titer of I-I-R was significantly higher than that of I-I-A (176 vs 93, 1.9-fold) (Fig. 1B). These results showed that although I-I-M had higher levels of anti-S IgG and WT Nab titers than I-I-A, the Nab titers did not differ significantly among the three groups for the remaining variants.

### I-I-M, I-I-A, and I-I-R exhibited comparable levels of RBD^+^ B-cell activation

**Fig 2:**
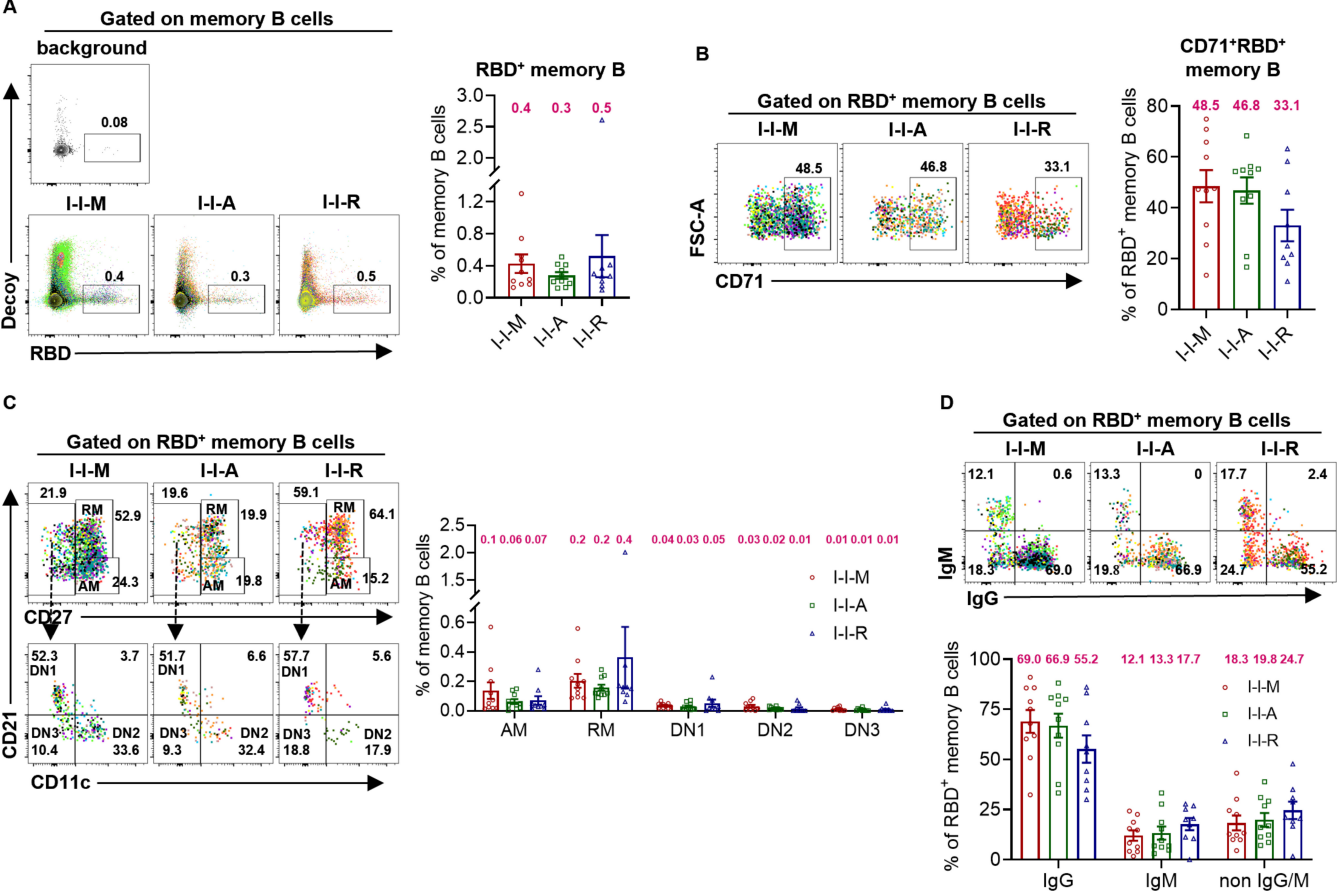
RBD^+^ memory B cell phenotypes among the I-I-M, I-I-A, and I-I-R groups. (A–B) The proportion of RBD^+^ memory B cells and CD71^+^ RBD^+^ memory B cells among I-I-M, I-I-A, and I-I-R. (C) The frequency of activated memory (AM), resting memory (RM), or double-negative (DN) subsets among I-I-M, I-I-A, and I-I-R. (D) The frequency of IgG^+^, IgM^+^ and non IgG/M^+^ RBD^+^ memory B cells among I-I-M, I-I-A, and I-I-R. The number in magenta indicates the mean in the frequency of detected responses, and each dot represents one donor (A–D). Significance was measured using the Mann‒Whitney test.

To further investigate the effects of the third dose vaccines on human SARS-CoV-2-specific B cells and elucidate the similarities and differences in Nab titers, we used a WT RBD probe to capture RBD^+^ B cells in the three groups and detected their related phenotypes using flow cytometry to analyze the activation status and isotype usage distribution of the RBD^+^ B cells. First, we found that the proportions of RBD^+^ memory B cells among I-I-M, I-I-A, and I-I-R were comparable (0.4, 0.3, and 0.5, respectively) (Fig. 2A). The RBD^+^ memory B cell-activating abilities of the different types of vaccines varied. The proportion of I-I-R CD71^+^ RBD^+^ memory B cells was lower than that in the other two groups (48.5, 46.8, and 33.1) (Fig. 2B). Then, to investigate whether the different types of vaccine can affect the RBD^+^ memory B-cell differentiation without changing the cell quantities, we examined the expression of CD11c, CD21, and CD27 on RBD^+^ memory B cells. Similarly, the proportion of resting memory B cells (RM, CD27^+^ CD21^+^), activated memory B cells (AM, CD27^+^ CD21^-^), double-negative B cells-1 (DN1, CD27^-^ CD11c^-^ CD21^+^), double-negative B cells-2 (DN2, CD27^-^ CD11c^+^ CD21^-^), and double-negative B cells-3 (DN3, CD27^-^ CD11c^-^ CD21^-^) did not differ among the three groups (Fig. 2C), suggesting that the mRNA, adenoviral vector, and recombinant protein vaccines showed comparable abilities to activate pre-existing RBD^+^ memory B cells. In the isotype usage division, the RBD^+^ memory B cells of I-I-M, I-I-A, and I-I-R were frequently classified as the IgG subtype (69.0%, 66.9%, and 55.2%, respectively) (Fig. 2D).

In summary, the activation and division of RBD^+^ memory B cells induced by the third dose vaccines did not differ significantly.

### IGHV1-69, IGHV3-9, and IGHV4-34 were the dominant immunoglobulin heavy chain variable (IGHV) regions of I-I-M, I-I-A, and I-I-R, respectively

**Fig 3:**
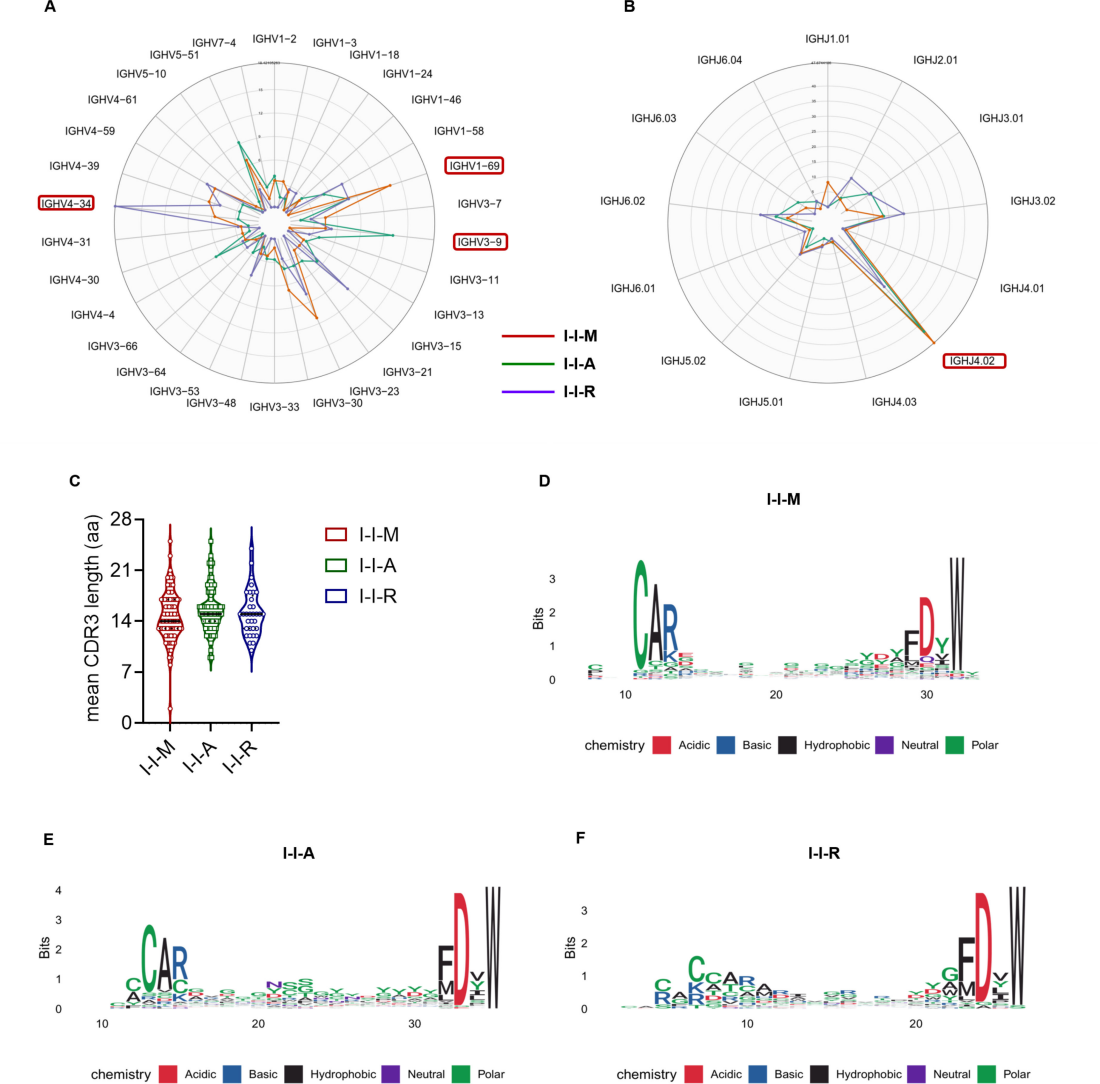
BCR sequencing analyses of the I-I-M, I-I-A, and I-I-R groups. (A–B) Radar plots show the usage of IGHV (**A**) and IGHJ (**B**) genotype in group I-I-M (red line), I-I-A (green line), and I-I-R (blue line). The values for each circle were labeled, which represent the proportion of given IGHV(J) genotype in given BCR repertoire. The minimum was set to zero, which indicates that no given genotype exists. (C) The length of CDR3 regions were counted for each BCR in each group of I-I-M, I-I-A, and I-I-R. The distribution of the CDR3 length in three groups was shown in different colored lines as legend pointed. (D–F) Sequence logo visualization of the V(D)J sequence alignments within each group of I-I-M (**D**), I-I-A (**E**), and I-I-R (**F**). The height of each letter indicates the usage of amino acid in each site.

The BCR is an important B-cell surface protein that recognizes specific antigens, and its species diversity determines the diversity of antibodies secreted by the B cells. Therefore, analyzing the BCR IGHV usage, length, and amino acid diversity of I-I-M, I-I-A, and I-I-R might be beneficial for further understanding the similarities and differences in humoral immune responses induced by the three immunization strategies. Because *IGHV* encodes the vast majority of regions that interact with the pathogen on the BCR (HCDR1, HCDR2, and partially HCDR3), we used single-cell BCR sequencing technology to investigate the similarities and differences in the BCR IGHV sequences of I-I-M, I-I-A, and I-I-R RBD^+^ B cells, and also to reveal the preference of the three immunization strategies for the predominant IGHV usage. First, we obtained 77, 89, and 38 effective IGHV sequences from I-I-M, I-I-A, and I-I-R, of which the IGHV compositions differ. Among them, I-I-M detected 25 IGHV sequences, with IGHV1-69 as the main component, accounting for 13.5% of the sequences; I-I-A detected 27 IGHV sequences, with IGHV3-9 as the main component, accounting for 13.2% of the sequences; 18 IGHV sequences were detected in I-I-R, and IGHV4-34 was the main component, accounting for 18.4% of the sequences (Fig. 3A). Moreover, using the Simpson index to test the clonal diversity, we found no significant IGHV diversity among the three groups (I-I-M: 0.938; I-I-A: 0.943 and I-I-R: 0.918). Unlike the diversity of IGHV, IGHJ usage in I-I-M, I-I-A, and I-I-R was relatively low (Simpson index: 0.733, 0.764, and 0.842, respectively), and the dominant usage was that of IGHJ4.02 (46.7%, 42.7%, and 22.9%, respectively) (Fig. 3B).

CDR3 is not only a variable fragment on the BCR but also an important region that determines the specificity of BCR recognition. Moreover, the insertion and deletion of the VD and DJ junction sequence segments result in high variation in the amino acid content and length of CDR3. The differential features of CDR3 sequences in BCR libraries from different cohorts possibly indicated a preference for sequence features unique to a class of antigens (18). No significant differences in the lengths of the three groups were observed (Fig. 3C; Fig. S2A). Further analysis showed that the amino acid sequences of the VD and DJ junctions of I-I-M, I-I-A, and I-I-R were not identical, but that the diversity of the amino acid species differed (Fig. 3D through F; Fig. S2B). This results in differences in the properties of RBD-specific antibodies. In summary, although the CDR3 length did not vary among the three BCR populations, the IGHV usage and amino acid species at the VD-DJ junction differed, further suggesting that the SARS-CoV-2-specific antibody profiles induced by the third dose vaccines differed in terms of antibody species and number.

### I-I-R group possessed large numbers of regulatory T cells (Treg), cTFH1, cTFH17, cTFH-CM cells

**Fig 4:**
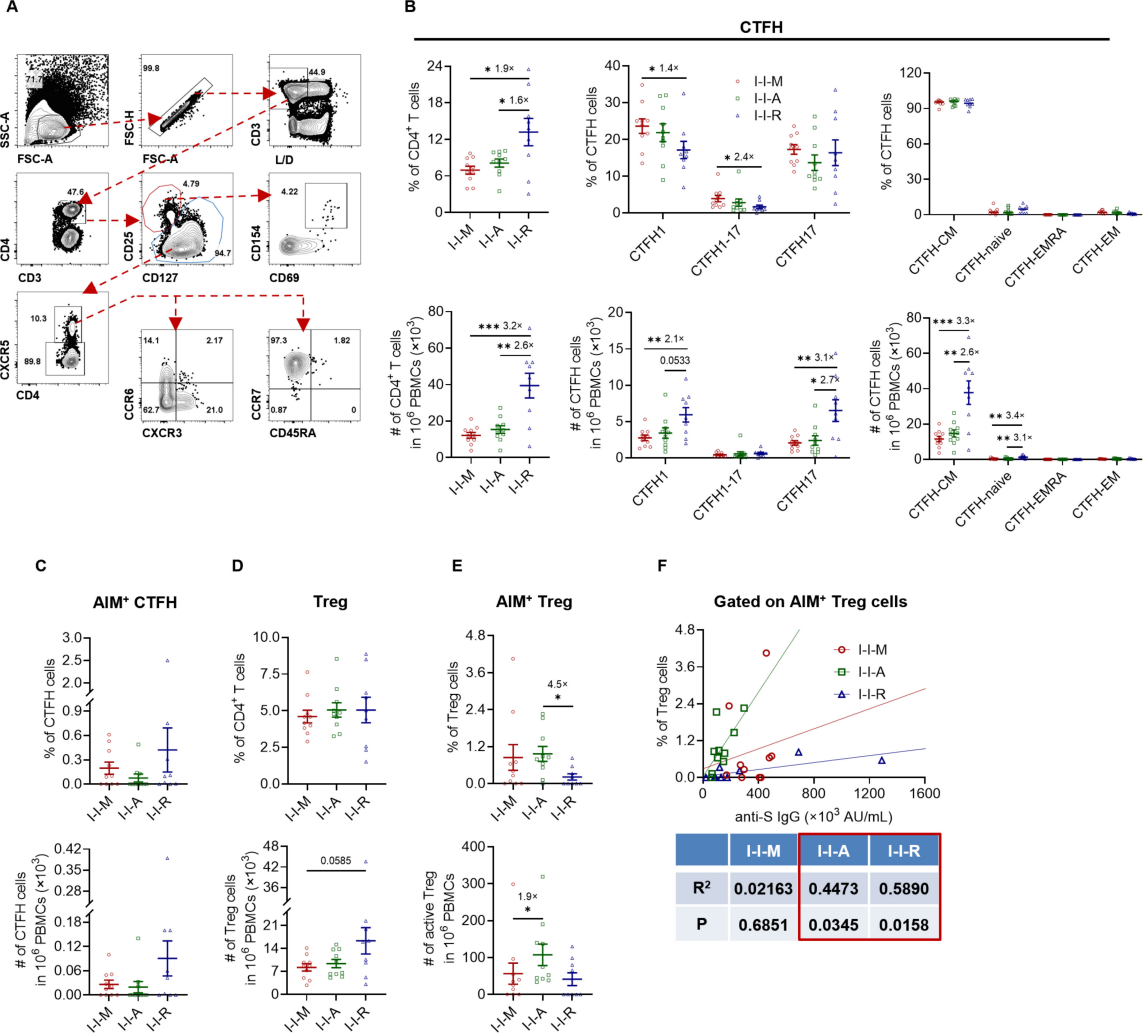
Frequency and numbers of cTFH and Treg cells among the I-I-M, I-I-A, and I-I-R groups. (A) Flow cytometry gating strategy for cTFH and Treg. (B) The frequency and number of bulk cTFH with different phenotypes among I-I-M, I-I-A, and I-I-R. (C) The frequency and number of AIM^+^ (CD69^+^CD154^+^) cTFH among I-I-M, I-I-A, and I-I-R. (D–E) The frequency and number of bulk and AIM^+^ (CD69^+^CD154^+^) Treg among I-I-M, I-I-A, and I-I-R. (F) Correlation of anti-S IgG titers and proportion of AIM^+^ Treg cells among I-I-M, I-I-A, and I-I-R, respectively. Fold change with significant difference was shown in (B–E). Significance was measured using the Mann‒Whitney test. *P < 0.05, **P < 0.01, ***P < 0.001.

Many studies have shown that immunization with SARS-CoV-2 vaccines induces not only a humoral immune response but also T-cell immune responses. Different types of T cells can also affect the humoral immune response. To evaluate the effects of the third dose vaccines on the differentiation, function, and proliferative ability of SARS-CoV-2-specific T cells, we used *ex vivo* stimulation and flow cytometry to detect the proportion and number of different T-cell populations and the expression of related cytokines, respectively. The results showed that the proportion of bulk cTFH in I-I-R was 1.9-fold and 1.6-fold of that in I-I-M and I-I-A, respectively, and that the cell numbers were 3.2-fold and 2.6-fold of that of I-I-M and I-I-A, respectively (Fig. 4A and B). Furthermore, we used two pairs of surface markers, CXCR3-CCR6 and CD45RA-CCR7, to finely divide the populations of cTFH1 (CXRC3^+^CCR6^-^), cTFH1-17 (CXRC3^+^CCR6^+^), cTFH17 (CXRC3^-^CCR6^+^), cTFH-CM (CD45RA^-^CCR7^+^), cTFH-naïve (CD45RA^+^CCR7^+^), cTFH-EMRA (CD45RA^+^CCR7^-^), and cTFH-EM (CD45RA^-^CCR7^-^). Although the proportions of cTFH1 and cTFH1-17 in I-I-M were higher than those in I-I-R (1.4-fold), the numbers of cTFH1 and cTFH17 in I-I-R were higher than those in the other two groups. The numbers of cTFH-CM and cTFH-naïve cells in the I-I-R group were higher than those of the other two groups. However, the proportion and number of activation-induced marker (AIM) (CD69^+^CD154^+^)-specific cTFH did not differ significantly among the three groups (Fig. 4C).

Tregs were also analyzed to some extent. Although no significant differences in the proportion and number of Tregs at the bulk level were observed, the AIM^+^ Treg population in the I-I-A group was proportionally higher than that in the I-I-R group and quantitatively higher than that in the I-I-M group (Fig. 4D and E). Furthermore, we found that the anti-S IgG antibody titers of I-I-A and I-I-R correlated with their respective proportions of AIM^+^ Treg cells (Fig. 4F), which may be related to the ability of adenoviral vector vaccines and recombinant protein vaccines to induce S-specific Treg production, further suggesting an association between S-specific Treg responses and humoral immune responses.

### I-I-A showed higher proportion and numbers of IFN-γ^+^ and IFN-γ^+^TNF^+^ CD4^+^ T cells than I-I-R *ex vivo*

**Fig 5:**
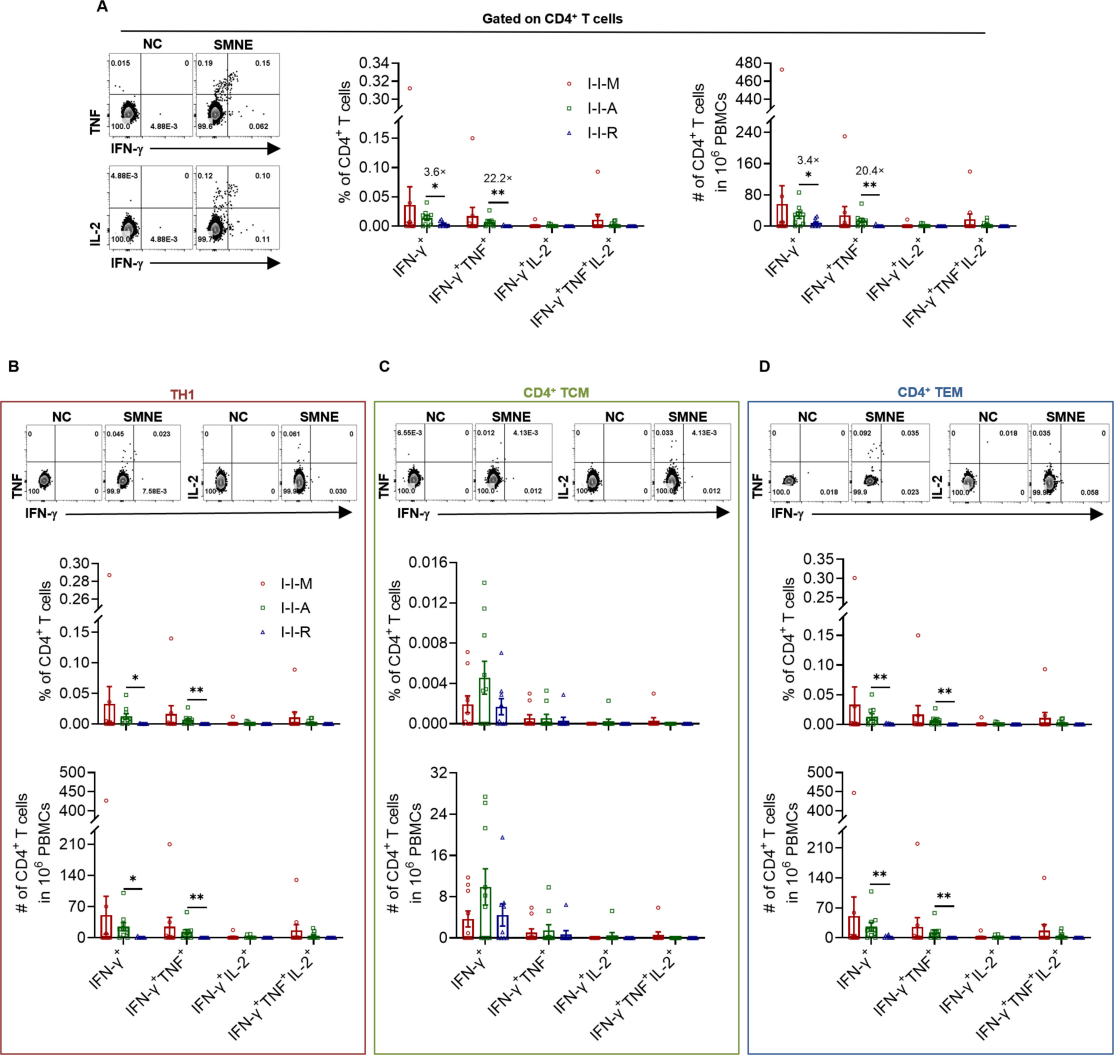
The cytokine secretion capacity of total CD4^+^ T cells, TH1, TH17, and TH1-17 among I-I-M, I-I-A, and I-I-R with *ex vivo* culture. (A–D) The proportion and numbers of IFN-γ^+^, IFN-γ^+^TNF^+^, IFN-γ^+^IL-2^+^ and IFN-γ^+^TNF^+^IL-2^+^ in total CD4^+^ T cells, TH1, TCM and TEM among I-I-M, I-I-A ,and I-I-R, respectively. Fold change with significant difference was shown in (A–D). Significance was measured using the Mann‒Whitney test. *P < 0.05, **P < 0.01, ***P < 0.001.

The ability of the SARS-CoV-2 vaccine to induce cytokine secretion by human SARS-CoV-2-specific T cells is also an important indicator of vaccine potency. For this purpose, we used *ex vivo* stimulation and flow cytometry to detect the ability of SARS-CoV-2 SMNE^+^ CD4^+^ T cells to secrete IFN-γ, TNF, and IL-2 and SMNE^+^ CD8^+^ T cells to secrete IFN-γ, TNF, and granzyme B (Grz B) in the three groups. Moreover, the ability of different TH cells and memory T cells to secrete cytokines was investigated to identify the SARS-CoV-2-specific T cell subtypes that play immune-dominant roles. SMNE^+^ IFN-γ^+^ and IFN-γ^+^TNF^+^ CD4^+^ T cells were detected in I-I-A (3.6-fold and 22.2-fold, respectively), and the cell numbers (3.4-fold and 20.4-fold, respectively) were significantly higher than those in I-I-R (Fig. 5A). No significant differences in the cytokine expression profiles of the SMNE^+^ CD8^+^ T cells of the three groups were observed, and the response intensity and multifunctionality of the SMNE^+^ CD8^+^ T cells were lower than those of SMNE^+^ CD4^+^ T cells (Fig. 5A; Fig. S3A).

To investigate the differences in the cytokine secretion abilities of SMNE^+^ TH1, TH1-17, and TH17 cells induced by different types of SARS-CoV-2 vaccines, we detected the expression of IFN-γ, TNF, and IL-2 in the corresponding cells of the three groups. The results showed that TH1 cells mainly secreted the three cytokines in all three groups, and that the responses of TH1-17 and TH17 cells decreased (Fig. S4A and B). Among them, I-I-A showed a higher proportion and number of IFN-γ^+^ and IFN-γ^+^TNF^+^ TH1 than I-I-R (Fig. 5B).

We also tested SMNE^+^ T cells of different memory types for their ability to secrete IFN-γ, TNF, and IL-2. A comparison of memory CD4^+^ T cells showed that the three groups mainly secreted IFN-γ, TNF, and IL-2 (Fig. 5C and D; Fig. S4C and D). Further comparison of cytokine intensities showed similar responses to total CD4^+^ T cells and TH1 cells; the proportion and quantity of IFN^-^γ^+^ and IFN-γ^+^TNF^+^ CD4^+^ effector memory T cells (TEM) in I-I-A was higher than that in I-I-R (Fig. 5D). In contrast, cytokine expression ability did not differ significantly among the different types of CD8^+^ T memory cells in the three groups, whereas the predominant SMNE^+^ CD8^+^ T cells were IFN-γ^+^ (Fig. S3B through E).

The above results showed that all three groups had a certain degree of SMNE^+^ CD4^+^ T cell response and mainly secreted cytokines via TH1 and CD4^+^ TEM. Furthermore, the proportion and quantity of IFN-γ^+^ and IFN-γ^+^TNF^+^ TH1 or CD4^+^ TEM cells in I-I-A were significantly higher than those in I-I-R.

### SMNE^+^ T cells in the I-I-R group exhibited low proliferative capacity

**Fig 6:**
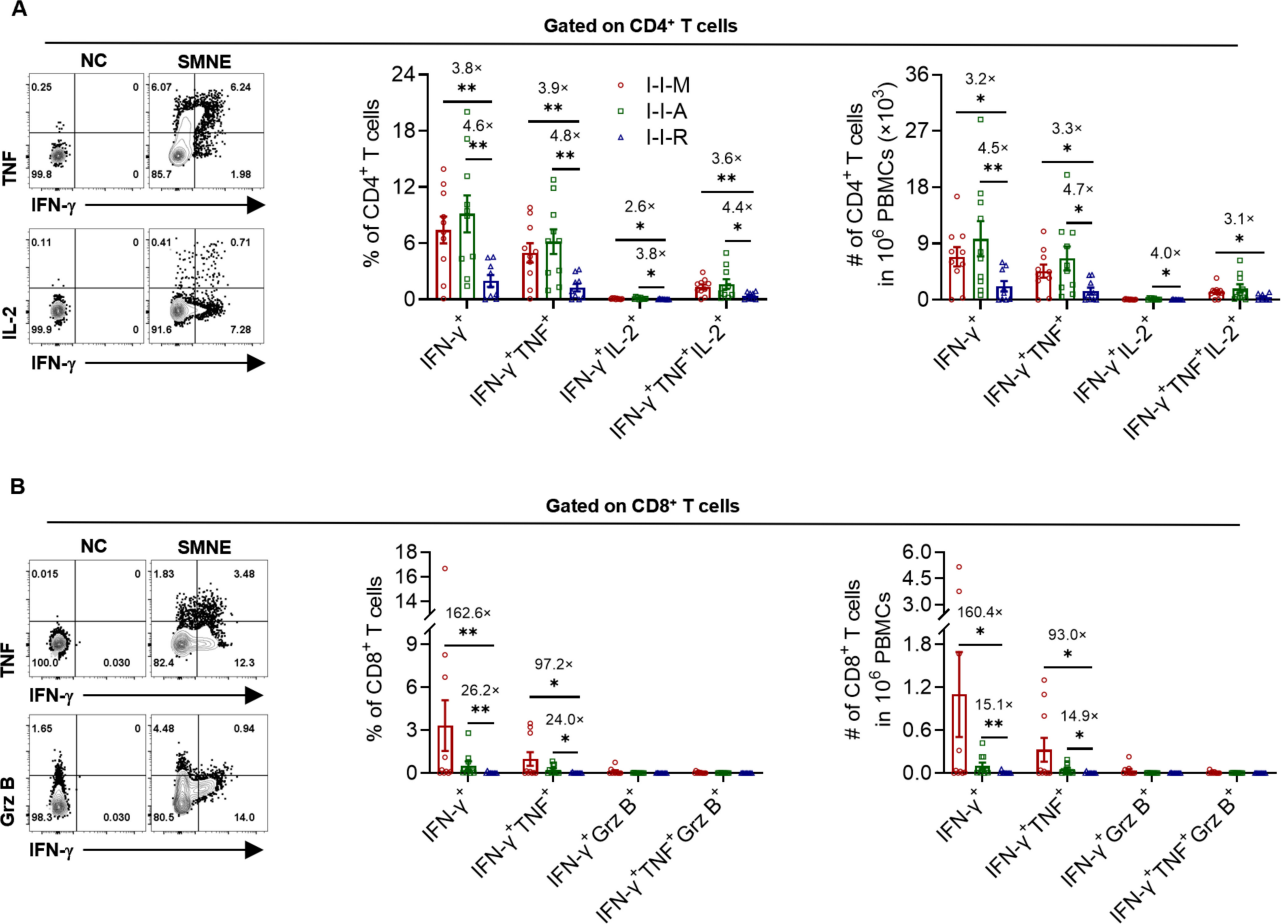
The cytokine secretion capacity of CD4^+^ and CD8^+^ T cells among I-I-M, I-I-A, and I-I-R with *in vitro* culture. (A) The proportion and numbers of IFN-γ^+^, IFN-γ^+^TNF^+^, IFN-γ^+^IL-2^+^ and IFN-γ^+^TNF^+^IL-2^+^ in total CD4^+^ T cells among I-I-M, I-I-A, and I-I-R. (B) The proportion and numbers of IFN-γ^+^, IFN-γ^+^TNF^+^, IFN-γ^+^grz B^+^, and IFN-γ^+^TNF^+^ grz B^+^ in total CD8^+^ T cells among I-I-M, I-I-A, and I-I-R. Fold change with significant difference was shown in (**A-B)**. Significance was measured using the Mann‒Whitney test. *P < 0.05, **P < 0.01, ***P < 0.001.

T cells undergo clonal proliferation following antigen stimulation. To assess the proliferative capacity of SMNE^+^ T cells induced by the third dose vaccines, we used a 10-day *in vitro* culture to specifically expand SMNE^+^ T cells from the cohort population samples and detected changes in the expression of relevant cytokines. The results showed that the SMNE^+^ CD4^+^ T cells of I-I-R could not be expanded to levels comparable to those of the other groups. The proportions and numbers of IFN-γ^+^, IFN-γ^+^TNF^+^, IFN-γ^+^IL-2^+^, and IFN-γ^+^TNF^+^IL-2^+^ cells in I-I-R were significantly lower than those in I-I-M and I-I-A (Fig. 6A). In contrast, I-I-M and I-I-A showed comparable cytokine expression intensity and multifunctionality (Fig. 6A). The proportion and quantity of IFN-γ^+^ and IFN-γ^+^TNF^+^ CD8^+^ T cells of the I-I-M and I-I-A groups expanded to some extent and showed stronger responses than those of the I-I-R group. However, the proportion and numbers of IFN-γ^+^Grz B^+^ and IFN-γ^+^TNF^+^Grz B^+^ CD8^+^ T cells did not change significantly (Fig. 6B). This further suggested that inactivated vaccines administered to the population were not effective in inducing memory SMNE^+^ CD8^+^ T cells, particularly those with multifunctional memory SMNE^+^ CD8^+^ T cells, whereas the mRNA, adenoviral vector, and recombinant protein vaccines showed weak ability to induce the SMNE^+^ CD8^+^ T cell response.

## DISCUSSION

The COVID-19 vaccine can induce Nabs to prevent SARS-CoV-2 infection; however, vaccine efficacy may be weakened by the mutations in the RBD (19). Therefore, the neutralization abilities of different COVID-19 vaccines against different SARS-CoV-2 variants should be evaluated. Some studies have reported the immune efficacy of different types of COVID-19 vaccines from various brands using different cohorts worldwide (11), which may have been influenced by factors such as ethnic differences, immune backgrounds, and epidemic prevention policies. Thus, the immune efficacy in the same cohorts should be evaluated using different types of COVID-19 vaccines from the same brand.

In this study, we found that although the anti-S IgG titer and Nab titers against WT of I-I-M was higher than that of I-I-A, the neutralizing abilities of Delta, BA.1, BA.2, and BA.5 were comparable to the other two groups, showing comparable SARS-CoV-2 variant-neutralizing abilities induced by mRNA, adenoviral, and recombinant protein vaccines. The differences were observed in the level of anti-S-IgG instead of Nabs among the three groups, considering that the binding antibodies can induce the ADCC protection, it will be interesting to investigate whether it will be better to choose those vaccines with high induction of binding antibodies, such as anti-S IgG upon facing the threat of the vaccine-candidate strain mismatched subvariants.

In addition, to further investigate the differences in the humoral immune responses elicited by the third dose vaccines, we examined the phenotypes of RBD^+^ memory B cells among I-I-M, I-I-A, and I-I-R. The RBD^+^ B cell activation and isotype usages of the three groups were similar, indicating that B-cell differentiation, as characterized by memory phenotype and isotype usage, was not influenced by the antigen encounter time points, at least not at day 14 after the vaccination. The frequency of BCR IGHV usage in the three groups was compared to determine the relationship between Nab titers and IGHV. We found that the dominant IGHVs were IGHV1-69 and IGHV3-23 in I-I-M, IGHV3-9 in I-I-A, and IGHV4-34 in I-I-R. However, previous studies have shown that IGHV1-69 and IGHV3-23 were predominantly detected in the primary and secondary WT-infected populations (20). Although the proportion of the primary positive population was higher, the frequency of IGHV usage in the secondary infected population showed significant averaging, with an increased proportion of IGHV1-2 (21). IGHV1-69 showed a strong ability to recognize the L452R mutation in different variants. Consequently, although IGHV1-69 weakly neutralizes the Delta and BA.5 variants containing the L452R mutation, it strongly neutralizes the BA.1 and BA.2 variants lacking the L452R mutation (22). IGHV3-23 is the main type of IGHV used in early recoverees infected with the WT virus; in addition, it can recognize Beta, Delta^+^ (delta mutant with K417N), and BA.1 variants with the N417 mutation, displaying a strong neutralizing effect on Beta and Delta^+^ variants, albeit showing slightly weaker neutralization of the BA.1 variants (23, 24). Thus, the differences in BCR usage induced by the third dose vaccines resulted in differences in protection against different existing variants, and may lead different protection efficacy against the future SARS-CoV-2 variants. Future studies should focus on pairing the relevant BCR and determining the recognition site of the corresponding antibody.

Study has shown that Middle East respiratory syndrome-CoV-specific memory T cells are characterized by longer survival, faster response, and recognition of more conserved epitopes of viral proteins, compared with neutralizing antibodies (25). Studies on SARS-CoV-2-specific T cells have shown that virus-specific memory T cells induced by vaccine immunization are influenced by antigenic modification and presentation, and the adjuvants used in different types of vaccines, which elicit different T-cell responses and differentiation, as well as immunoinflammatory conditions (26). Therefore, the phenotype, function, and proliferation of SARS-CoV-2-specific T cells induced by second-generation SARS-CoV-2 vaccines, and their correlation with the humoral immune response, are of considerable concern. First, our results showed that the bulk cTFH in I-I-R was significantly higher than those in I-I-M and I-I-A, probably because the recombinant protein is soluble and activates cTFH and TH2 (27), whereas mRNA or adenovirus is taken up by antigen-presenting cells, inducing TH1 and cytotoxic T lymphocyte responses. The SARS-CoV-2-specific AIM^+^ cTfh and Treg did not show significant differences in the proportion and number of SARS-CoV-2 specific cTFH among the three groups, which was consistent with the trend in antibody levels. Interestingly, the proportion of AIM^+^ Tregs in I-I-A and I-I-R correlated with anti-S IgG titers, suggesting the potential role of Tregs in inhibiting the activation of the remaining virus-specific T cells to some extent and slowing down the rate of antigen clearance, thereby prolonging the engagement of antigens with memory B cells and resulting in increased antibody expression. However, this mechanism remains to be investigated. Additionally, we performed cytokine expression and proliferation assays using different types of TH and memory T cells. The third dose vaccines induced TH1 and CD4^+^ TEM cells to express IFN-γ, TNF, and IL-2, which is consistent with previously reported data (28, 29). However, in both *ex vivo* and *in vitro* assays, the SMNE-specific CD4^+^ T cell abilities in the I-I-R group, such as cytokine expression, multifunction, differentiation, and proliferation, were considerably lower than those in the I-I-M and I-I-A groups. This may be because of antigen modification and presentation,

In summary, our study not only measured the magnitude and breadth of neutralizing antibodies of different vaccination strategies, but also further interpret their underlying mechanism, including BCR usage and T cell differentiation and bias. It will help us understand the antigen epitope hierarchy induced by different vaccine strategies. In addition, the strong SARS-CoV-2-specific CD4^+^ T cell response in the I-I-M and I-I-A groups also suggested that I-I-M and I-I-A sequential immunization strategies are effective for inducing cellular immune responses, indicating these two vaccination strategies are better in inducing T-B immune balance against SARS-CoV-2 infection. However, our study did not show whether the difference of BCR usage is derived from vaccine antigen design and sequence modification, delivery format/route, adjuvants types, or antigen duration. Therefore, future large cohorts and detailed studies are needed to completely reflect the similarities and differences in SARS-CoV-2-specific immune responses induced by the third dose vaccines.

## MATERIALS AND METHODS

### Cohort and sample preparation

This study recruited 10 people receiving one dose of mRNA vaccine 14 days after two doses of inactivated vaccine (I-I-M, *n* = 10), 10 people receiving one dose of Adv vector vaccine 14 days after two doses of inactivated vaccine (I-I-A, *n* = 10) and nine people who were vaccinated with one dose of recombinant protein vaccine for 14 days after two doses of inactivated vaccine (I-I-R, *n* = 9) (Table S1). This study was approved and supervised by the GMUH Ethics Committee (No.2021–78). Written informed consent was obtained from all the enrolled volunteers. Plasma and peripheral blood mononuclear cells (PBMCs) were prepared as previously described (30). Briefly, whole blood was collected into sodium heparin and EDTA tubes by standard phlebotomy. Plasma was separated by centrifuging blood tubes at 800*g* for 10 min, and aliquots were stored at −80°C for subsequent antibody analyses. The remaining cellular fraction was diluted with an equal volume of DPBS containing 2% FBS and 1% P/S. Then, the PBMCs were isolated from heparinized whole blood by density-gradient sedimentation using Ficoll–Paque according to the manufacturer’s instructions (GE Healthcare, 17–1440-02).

### PBMC isolation and *ex vivo* stimulation

PBMCs were isolated from heparinized whole blood by density-gradient sedimentation using Ficoll–Paque according to the manufacturer’s instructions (GE Healthcare, 17–1440-02). The PBMCs (5 × 10^5^) were then cultured in complete RPMI (c-RPMI, RPMI 1640 medium (Gibco) enriched with supplements, including 10% heat-inactivated FBS (Biological Industries, Israel Beit-Haemek), 100 µM MEM nonessential amino acids (Gibco), 100 U/mL penicillin (Gibco), 0.1 mg/mL streptomycin (Gibco), 2 mM L-glutamine (Gibco), 25 mM HEPES (Gibco), 55  µM 2-mercaptoethanol (Gibco), and 1 mM sodium pyruvate (Gibco)). The PBMCs were treated with the peptide pool containing 487 15-mer peptides (250 nM of each peptide) in the presence of 10 U/mL rIL-2 and 1 µM GolgiPlug (BD Biosciences, San Diego, CA) for 16 h at 37 °C with 5% CO_2_. The approach of using a large peptide pool to stimulate PBMCs was based on that developed by Chevalier et al. (31) and was validated.

### ELISA for detecting anti-S IgG antibody clusters

To detect specific plasma anti-S IgG antibody titers in the vaccination samples that were collected from heparinized whole blood by centrifugation at 800 *g* for 10 min. A sample with a high anti-S titer was used as the standard sample, with an arbitrary unit (unit/mL) of 2,000. Next, a two-fold serial dilution of the standard sample was performed at each instance for the evaluation of other plasma samples. A washing buffer was used as the blank control. After incubation with the plasma samples, Goat Anti-Human IgG Secondary Antibody (HRP) was added for 10 min at 37 °C. Absorbance was measured at 450 nM by a Multiskan GO microplate spectrophotometer (Thermo Fisher Scientific, Waltham, MA, USA). Data were analyzed using a standard curve with a log-logistic model.

### SARS-CoV-2 conventional virus neutralization test

A neutralization test for WH-1 (wild-type SARS-CoV-2) and VOCs, including B.1.617.2 (Delta) and BA.1, BA.2, and BA.4/5, was performed in a certified BSL-3 laboratory, as previously described (32). Fifty microliters of plasma sample was serially diluted, mixed with 50 µL of virus (100 TCID50) in 96-well flat-bottom plates, and incubated for 1 h at 37 °C. VERO E6 cells (1.2 × 10^4^, ATCC, USA) were seeded in these mixtures and incubated at 37 °C for 4 days; the cytopathic effect was examined using a Celigo Imaging Cytometer.

### RNA extraction and reverse transcription

The procedure for PCR amplification has been described elsewhere (33, 34). Briefly, total RNA from each RBD^+^ memory B cell lysate was subjected to reverse transcription with SuperScript IV VILO Master Mix (Thermo Fisher) at 25 °C for 10 min, 42 °C for 120 min, 85 °C for 5 min. The resulting cDNA was used as a template for amplifying the heavy and light chains with PrimeSTAR Max DNA Polymerase (Takara) and V gene-specific primer mixes (Efficient generation of monoclonal antibodies from single human B cells by single-cell RT-PCR and expression vector cloning), in a sequential seminested approach. PCR products were analyzed by gel electrophoresis to check that the bands obtained were of the correct size, and were then subjected to Sanger sequencing. The sequences obtained were analyzed with IMGT, the international ImMunoGeneTics information system (http://www.imgt.org), to identify the V(D)J gene segments with the highest identity and the numbers of SHMs in VH genes.

### The IGH sequence analysis

The IGHV(J) information were collected according to the sequencing results of each sample sets (I-I-M, I-I-A, and I-I-R). The usage of each genotype in each group was calculated as the number of sequences with a given genotype divided by the total number of sequences. R package ggradar was used for visualization.

The V(D)J sequences were collected according to the sequencing results of each sample set (I-I-M, I-I-A, and I-I-R). ClustalW was performed with all sequences in each group. The aligned sequences were then sent for Logo Plot with R package ggseqlogo.

### Peptide pool design and preparation

SARS-CoV-2-specific peptides were designed and synthesized as follows. Each peptide was dissolved in DMSO and then pooled, with each at a concentration of 45 µM to form a stock. In total, 487 15-mer SARS-CoV-2 peptides (overlapping by 11 amino acids) spanning the entire antigen region of spike (S), membrane (M), nucleocapsid (N), and envelope (E) proteins were designed using an online peptide generator (Peptide 2.0) and synthesized by ChinaPeptides(QYAOBIO) (Shanghai) with a purity >80%.

### *In vitro* PBMC expansion, culture, and stimulation

As previously described (30), 5 × 10^5^ PBMCs were treated with the peptide pool (250 nM of each peptide) and incubated for 10 days for *in vitro* culture and stimulation. During this culture, half of the medium was replaced with fresh c-RPMI containing 10 U/ml rIL-2 twice per week. The cells were sub-cultured when needed. On day 10, the cells were restimulated 16 h with medium containing the peptide pool (250 nM of each peptide) before being stained for FACS analysis.

### WT SARS-CoV-2-specific memory B cell analyses

Antigen-specific B cells were detected as previously described (35). Biotinylated proteins were multimerized by mixing with fluorescently labeled streptavidin (SA) and incubated for 1 h at 4 °C as follows: WT Spike RBD and SA-BB515 at a mass ratio of 2:1 (25 ng RBD with 12.5 ng SA; ~4:1 molar ratio). SA-APC-Cy7 was not multimerized with biotinylated protein and was used as a decoy probe to gate out cells that non-specifically bind streptavidin. Antigen probes for RBD were multimerized before each stain. Total B cells from 5 × 10^6^ cryopreserved PBMCs were separated using the magnetic bead sorting, and the B cells were transferred to a 96-well U-bottom plate for surface staining as follows. First, cells were incubated with Human TruStain FcX (Fc receptor blocking solution, Biolegend, 1:200) and Fixable Viability Stain 440UV for 15 min at room temperature. Cells were then washed once with FACS buffer and stained with 75 µL antigen probe containing 25 ng RBD-BB515 and 20 ng SA-APC-Cy7 decoy for 1 h at 4 °C. The cells were then washed again and stained with anti-CD11c, anti-CD19, anti-CD20, anti-CD21, anti-CD27, anti-CD38, anti-CD71, anti-IgD, anti-IgM, and anti-IgG for 30 min at room temperature. The cells were then washed again and fixed for 20 min using 1% PFA in PBS. Gates were set using healthy donor samples stained without antigen probes and identical gates were used for all experimental runs.

### Detection of SARS-CoV-2-specific T cells

PBMCs were thawed by warming frozen cryovials in a 37°C water bath and resuspending cells in 10 mL of RPMI supplemented with c-RPMI. Cells were washed once in c-RPMI, counted using a Countess automated cell counter (Thermo Fisher), and resuspended in fresh c-RPMI to a density of 5 × 10^6^ cells/mL. For each condition, duplicate wells containing 5 × 10^5^ cells in 100 µL were plated in 96-well round-bottom plates and rested overnight in a humidified incubator at 37 °C, 5% CO_2_. After 16 h, CD40 blocking antibody (0.5 mg/mL final concentration) was added to cultures for 15 min before stimulation. The cells were then stimulated for 16 h with peptide pools (SMNE). At 12 h poststimulation, antibodies targeting CXCR3, CCR7, CD40L, CXCR5, and CCR6 were added to the culture along with GolgiPlug for a 4-h stain at 37 °C. After 4 h, duplicate wells were pooled, and the cells were washed in PBS. The cells were stained for 15 min at room temperature with Fixable Viability Stain 440UV and Fc receptor blocking solution (Human TruStain FcX, BioLegend) and washed once in FACS buffer. Surface staining for 30 min at room temperature was then performed with antibodies directed against CD3, CD4, CD8, CD25, CD45RA, CD69, and CD127 in FACS buffer. After fixation and permeabilization with Cytofix and Perm (BD Bioscience, Cat# 554714) on ice for 20 min, intracellular staining (ICS) was performed on ice for 30 min with anti-TNF, anti-IFN-γ, anti-IL-2, and anti-granzyme B and washed twice in perm/wash. After the final wash, the cells were resuspended in 300 µL FACS buffer.

### Flow cytometry

Flow cytometry data were collected on a BD Fortessa X-20 instrument. Standardized SPHERO rainbow beads (Spherotech) were used to track and adjust photomultiplier tubes over time. UltraComp eBeads (Thermo Fisher) were used for compensation. Up to 5 × 10^6^ cells were acquired per sample. Data were analyzed using FlowJo v10 (BD Bioscience).

### Statistical analysis

All statistical analyses were performed using GraphPad Prism software. Statistical significance was set at *P* < 0.05 (*P <  0.05; **P <  0.01; ***P <  0.001). Student’s t test was used to analyze differences in mean values between groups. The Mann‒Whitney test was used to compare the central tendencies of two groups (mean or median). Antibody responses are reported as geometric mean titers (GMT) with the 95% CI. The Wilcoxon rank-sum test was used to compare paired continuous variables not normally distributed. Cutoff values were assigned to evaluate the significance of the *P*-value according to the different statistical analysis methods indicated in each Figure legend. All values are presented as the mean ± SEM.

## SUPPLEMENTAL MATERIAL

Tables S1 mbio.01429-24-s0001.docx Demographics of 10 volunteers in I-I-M, 10 volunteers in I-I-A, and 9 volunteers in I-I-R.

Tables S2 mbio.01429-24-s0002.docx The Nab titers against WT, Delta, BA.1, BA.2, and BA.5 of 10 volunteers in I-I-M.

Tables S3 mbio.01429-24-s0003.docx The Nab titers against WT, Delta, BA.1, BA.2, and BA.5 of 10 volunteers in I-I-A.

Tables S4 mbio.01429-24-s0004.docx The Nab titers against WT, Delta, BA.1, BA.2, and BA.5 of 9 volunteers in I-I-R.

Fig. S1 mbio.01429-24-s0005.tif Comparison of anti-S IgG titers and Nab titers against WT, Delta, BA.1 BA.2, and BA.5 among I-I-M, I-I-A, and I-I-R.

Fig. S2 mbio.01429-24-s0006.tif CDR3 length and V(D)J sequence diversities of the I-I-M, I-I-A, and I-I-R groups.

Fig. S3 mbio.01429-24-s0007.tif The cytokine secretion capacity of total CD8+ T cells, TEM, Tnaive, TEMRA, and TEM among I-I-M, I-I-A, and I-I-R with *ex vivo* culture.

Fig. S4 mbio.01429-24-s0008.tif The cytokine secretion capacity of TH1-17, TH17, CD4+ Tnaive, and CD4+ TEMRA among I-I-M, I-I-A, and I-I-R with *ex vivo* culture.

ASM does not own the copyrights to Supplemental Material that may be linked to, or accessed through, an article. The authors have granted ASM a non-exclusive, world-wide license to publish the Supplemental Material files. Please contact the corresponding author directly for reuse.
