# Supplementary material for: Diverse BCR usage and T cell activation induced by different COVID-19 sequential vaccinations
Source: mBio. 2024 Sep 9;15(10):e01429-24. doi: 10.1128/mbio.01429-24 (PMC11481494; doi:10.1128/mbio.01429-24)
Supplement: Tables S1 — Demographics of 10 volunteers in I-I-M, 10 volunteers in I-I-A, and 9 volunteers in I-I-R. [file mbio.01429-24-s0001.docx]

**Supplementary Table 1: Demographics of 10 volunteers in I-I-M, 10 volunteers in I-I-A and 9 volunteers in I-I-R**

| Category | I-I-M (*n* = 10) | I-I-A (*n* = 10) | I-I-R (*n* = 9) |
| --- | --- | --- | --- |
| Age | 27.1  (23.1-31.1) | 29.5  (25.1-33.9) | 32.4  (28.0-36.9) |
| Gender |  |  |  |
| Female | 5 (50%) | 7 (70%) | 1 (11%) |
| Male | 5 (50%) | 3 (30%) | 8 (89%) |
| The interval between 2^nd^ and 3^rd^ vaccination (IQR) | 223.4  (142.5-304.4) | 305.6  (294.0-317.1) | 285.2  (262.9-307.6) |
| Days after the 3^rd^ vaccine inoculated | 14 | 14 | 14 |
| Vaccine | CoronaVac & RQ3013 | CoronaVac & ChAdTS-S | CoronaVac & ZR202-CoV |
