# Supplementary material for: Diverse BCR usage and T cell activation induced by different COVID-19 sequential vaccinations
Source: mBio. 2024 Sep 9;15(10):e01429-24. doi: 10.1128/mbio.01429-24 (PMC11481494; doi:10.1128/mbio.01429-24)
Supplement: Tables S2 — The Nab titers against WT, Delta, BA.1, BA.2, and BA.5 of 10 volunteers in I-I-M. [file mbio.01429-24-s0002.docx]

**Supplementary Table 2: The Nab titers against WT, Delta, BA.1, BA.2 and BA.5 of 10 volunteers in I-I-M**

| volunteers | WT | Delta | BA.1 | BA.2 | BA.5 |
| --- | --- | --- | --- | --- | --- |
| M1 | 384 | 256 | 64 | 192 | 32 |
| M2 | 512 | 256 | 32 | 64 | 96 |
| M3 | 1024 | 384 | 192 | 192 | 192 |
| M4 | 512 | 512 | 48 | 96 | 96 |
| M5 | 1024 | 768 | 128 | 128 | 96 |
| M6 | 1536 | 1024 | 256 | 256 | 768 |
| M7 | 1536 | 1536 | 192 | 256 | 384 |
| M8 | 768 | 768 | 192 | 128 | 128 |
| M9 | 1536 | 1024 | 384 | 768 | 192 |
| M10 | 1536 | 512 | 384 | 512 | 384 |
