# Supplementary material for: Diverse BCR usage and T cell activation induced by different COVID-19 sequential vaccinations
Source: mBio. 2024 Sep 9;15(10):e01429-24. doi: 10.1128/mbio.01429-24 (PMC11481494; doi:10.1128/mbio.01429-24)
Supplement: Tables S3 — The Nab titers against WT, Delta, BA.1, BA.2, and BA.5 of 10 volunteers in I-I-A. [file mbio.01429-24-s0003.docx]

**Supplementary Table 3: The Nab titers against WT, Delta, BA.1, BA.2 and BA.5 of 10 volunteers in I-I-A**

| volunteers | WT | Delta | BA.1 | BA.2 | BA.5 |
| --- | --- | --- | --- | --- | --- |
| A1 | 256 | 192 | 24 | 32 | 32 |
| A2 | 384 | 192 | 64 | 64 | 64 |
| A3 | 384 | 256 | 96 | 96 | 48 |
| A4 | 1536 | 768 | 128 | 192 | 96 |
| A5 | 128 | 64 | 16 | 32 | 32 |
| A6 | 512 | 512 | 192 | 256 | 192 |
| A7 | 512 | 64 | 384 | 192 | 384 |
| A8 | 512 | 256 | 24 | 64 | 48 |
| A9 | 1024 | 1536 | 192 | 96 | 96 |
| A10 | 768 | 1024 | 192 | 128 | 48 |
