# Supplementary material for: Diverse BCR usage and T cell activation induced by different COVID-19 sequential vaccinations
Source: mBio. 2024 Sep 9;15(10):e01429-24. doi: 10.1128/mbio.01429-24 (PMC11481494; doi:10.1128/mbio.01429-24)
Supplement: Tables S4 — The Nab titers against WT, Delta, BA.1, BA.2, and BA.5 of 9 volunteers in I-I-R. [file mbio.01429-24-s0004.docx]

**Supplementary Table 4: The Nab titers against WT, Delta, BA.1, BA.2 and BA.5 of 9 volunteers in I-I-R**

| volunteers | WT | Delta | BA.1 | BA.2 | BA.5 |
| --- | --- | --- | --- | --- | --- |
| R1 | 1024 | 1536 | 512 | 768 | 512 |
| R2 | 4 | 4 | 4 | 4 | 4 |
| R3 | 384 | 32 | 12 | 48 | 4 |
| R4 | 1024 | 768 | 128 | 768 | 384 |
| R5 | 1536 | 1024 | 512 | 1024 | 256 |
| R6 | 768 | 512 | 256 | 384 | 192 |
| R7 | 768 | 256 | 96 | 192 | 64 |
| R8 | 384 | 96 | 32 | 96 | 64 |
| R9 | 768 | 128 | 128 | 192 | 96 |
