# Supplementary figures and images for: Diverse BCR usage and T cell activation induced by different COVID-19 sequential vaccinations
Source: mBio. 2024 Sep 9;15(10):e01429-24. doi: 10.1128/mbio.01429-24 (PMC11481494; doi:10.1128/mbio.01429-24)

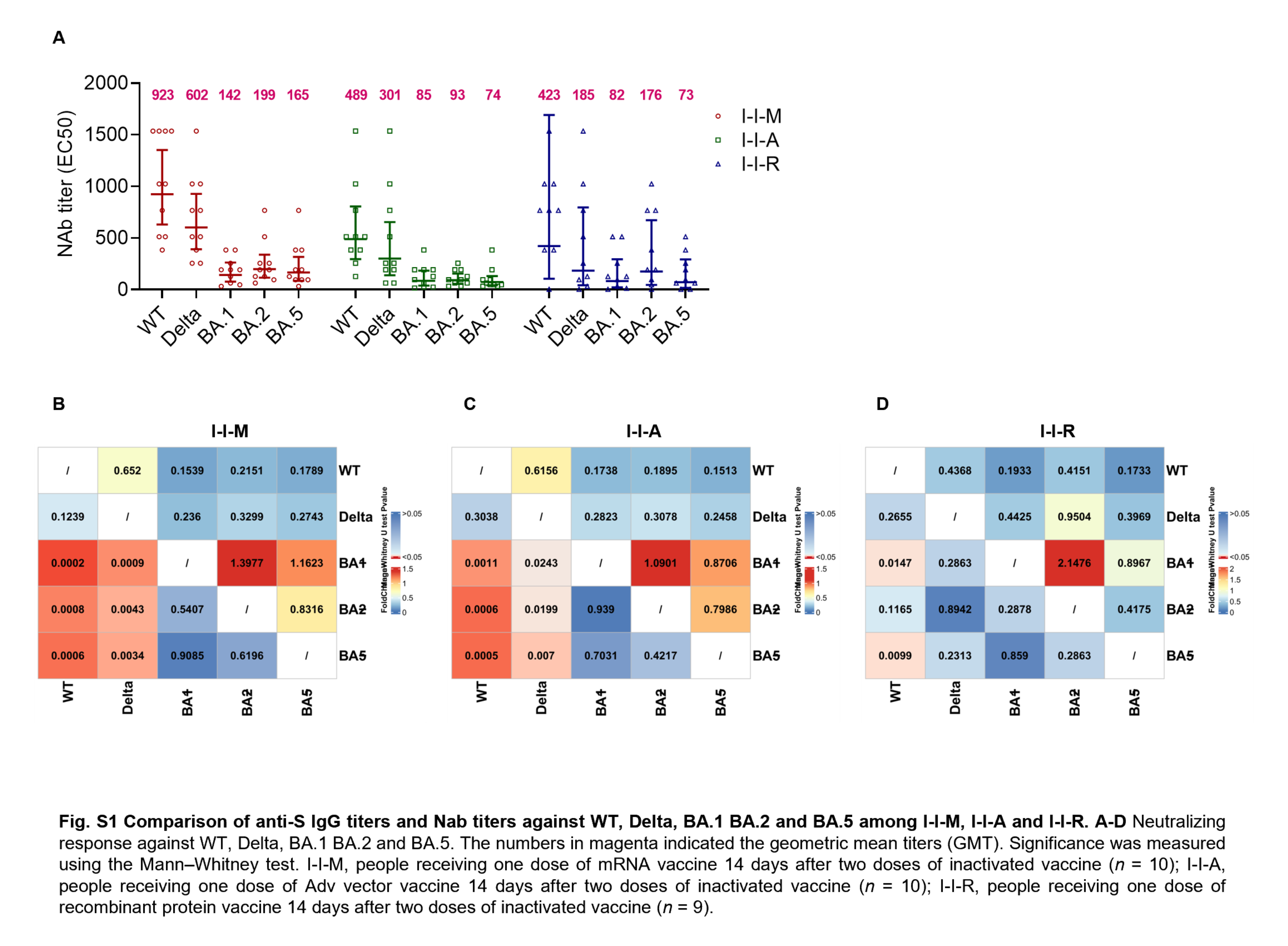

Supplement: Fig. S1 — Comparison of anti-S IgG titers and Nab titers against WT, Delta, BA.1 BA.2, and BA.5 among I-I-M, I-I-A, and I-I-R. [file mbio.01429-24-s0005.tif]

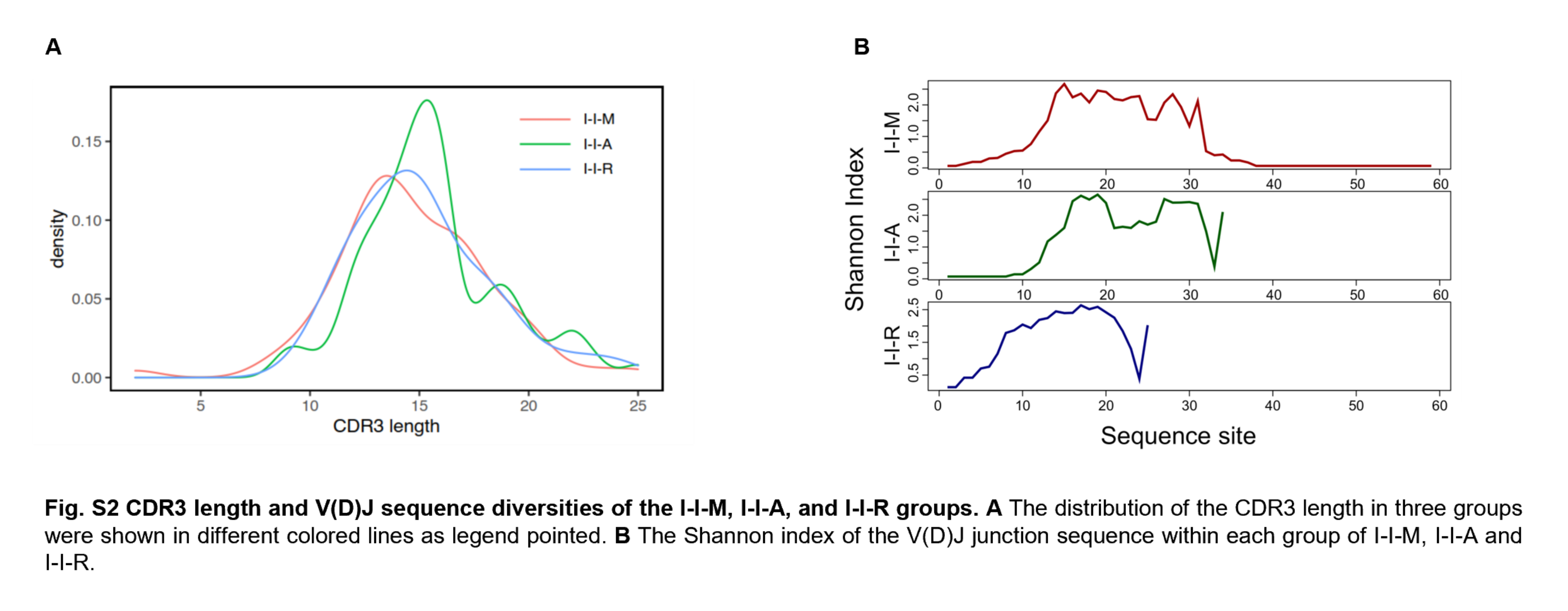

Supplement: Fig. S2 — CDR3 length and V(D)J sequence diversities of the I-I-M, I-I-A, and I-I-R groups. [file mbio.01429-24-s0006.tif]

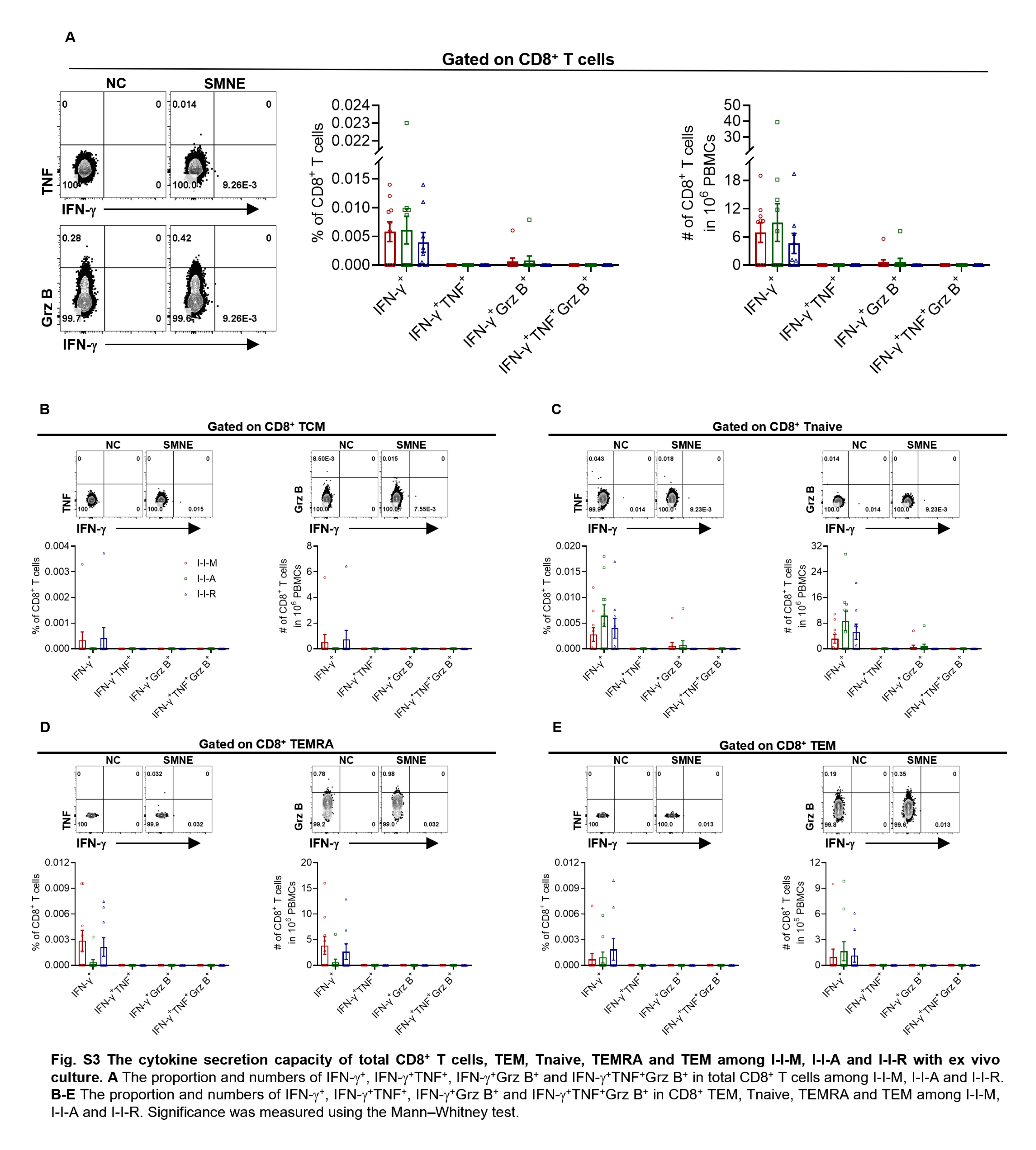

Supplement: Fig. S3 — The cytokine secretion capacity of total CD8+ T cells, TEM, Tnaive, TEMRA, and TEM among I-I-M, I-I-A, and I-I-R with ex vivo culture. [file mbio.01429-24-s0007.tif]

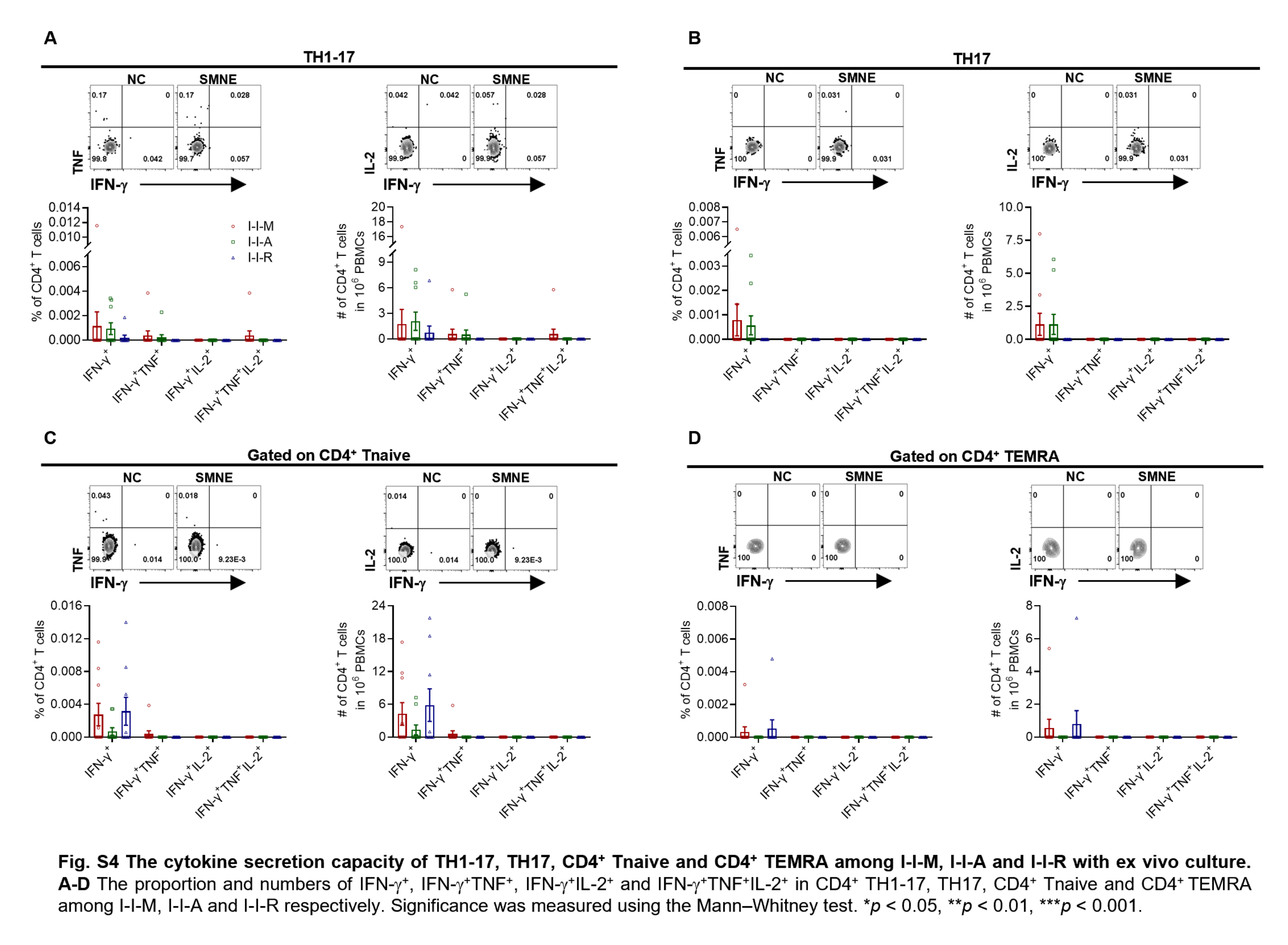

Supplement: Fig. S4 — The cytokine secretion capacity of TH1-17, TH17, CD4+ Tnaive, and CD4+ TEMRA among I-I-M, I-I-A, and I-I-R with ex vivo culture. [file mbio.01429-24-s0008.tif]
